# Comparison of three obturator nerve block techniques for injectate spread into the obturator canal: a randomized controlled trial

**DOI:** 10.1007/s00540-022-03055-6

**Published:** 2022-03-19

**Authors:** Tetsuya Uchino, Masahiro Miura, Shigekiyo Matsumoto, Chihiro Shingu, Toshitaka Shin, Kenichiro Tomonari, Takaaki Kitano

**Affiliations:** 1 Department of Anesthesiology, Faculty of Medicine, Oita University, 1-1, Idaigaoka, Hazamacho, Yufushi, Oita, 879-5503 Japan; 2 Department of Human Anatomy, Faculty of Medicine, Oita University, Oita, Japan; 3 Department of Urology, Faculty of Medicine, Oita University, Oita, Japan; 4 Oita Diagnostic Imaging Center, Oita, Japan

**Keywords:** Obturator nerve, Obturator canal, External obturator muscle, Obturator nerve block, Ultrasonography

## Abstract

**Purpose:**

The obturator nerve branches into the obturator canal; therefore, local anesthetic spread into the obturator canal predicts the success of the obturator nerve block (ONB). We compared three ONB techniques for the spread of local anesthetic mixed with contrast medium into the obturator canal.

**Methods:**

We performed the ONB using the classical pubic approach (PA), inguinal approach (IA), or ultrasound-guided methodologic approach (UMA) in 143 patients undergoing transurethral resection of bladder tumors. The obturator nerve course and branching patterns of the UMA group were examined using ultrasound imaging. After injecting a local anesthetic mixed with a contrast medium, we evaluated its spread into the obturator canal using fluoroscopic imaging. *P* < 0.05 indicated statistical significance.

**Results:**

Success rate of obturator canal enhancement was the greatest in the UMA group (84%; *P* < 0.001); the PA (42.6%; 20/47 patients) and IA (47.8%; 22/46 patients) groups did not differ significantly (*P* = 1.000). Both branches of the obturator nerve passed above the superior margin of the external obturator muscle (EOM), and the obturator canal was enhanced in 13 of 50 (26%) patients in the UMA group. The posterior branch of the obturator nerve passed between the superior and main fasciculi of the EOM in 37 of 50 patients (74%) in the UMA group; the obturator canal was enhanced in 29 of these 37 patients (78%).

**Conclusion:**

Local anesthetic spread into the obturator canal using the UMA was superior to that using the PA and IA. Both branches of the obturator nerve could be blocked using the UMA.

## Introduction

The obturator nerve block (ONB) is widely used for transurethral resection of bladder tumors (TURBT) to prevent unexpected adductor reflex [1], manage adductor muscle spasms [2], and ensure optimal analgesia for knee joint surgery [3–5]. The ONB using a nerve stimulator has been adapted in various ways [2, 6, 7]. Recently, ultrasound-guided ONB techniques have been widely adopted [8–10]. There are also reports of the use of ultrasound-guided proximal approaches to block both branches of the obturator nerve using an injection at a single site [11–15].

The obturator nerve divides into the anterior and posterior branches in the obturator canal. After passing through the obturator canal, these branches are first separated by some of the fasciculi of the obturator externus muscle; then, they are separated by the adductor brevis muscle, followed by formation of various branching patterns [16–18]. To efficiently block both branches of the obturator nerve, most proximal ONB techniques using a nerve stimulator target the obturator nerve that passes through the obturator canal [2, 6]. Therefore, we postulated that a single injection of the local anesthetic would be more likely to spread into the obturator canal immediately using the nerve stimulator-guided proximal technique compared to using the nerve stimulator-guided distal technique or the ultrasound-guided proximal technique.

This study compared the nerve stimulator-guided pubic approach (PA); inguinal approach (IA), performed at a more distal site than the site of the PA; and ultrasound-guided methodologic approach (UMA) for the spread of a mixture of local anesthetic and contrast medium into the obturator canal in patients undergoing TURBT. The primary outcome was the spread of the mixed solution into the obturator canal during each ONB. Secondary outcomes were complications such as vascular puncture and nerve damage.

## Methods

This was a randomized controlled study to assess the spread of injectate that was injected into the obturator canal using the following three approaches: PA, IA, or UMA. This study was approved by the Institutional Review Board of Oita University Faculty of Medicine (approval number: B11-043) and was registered with the University Hospital Medical Information Network Clinical Trials Registry (UMIN000008306; http://www.umin.ac.jp). All study participants provided written informed consent.

We enrolled 150 men with an American Society of Anesthesiologists physical status of I–III who underwent TURBT requiring ONB between August 2012 and December 2017. For patients who needed a bilateral ONB, the local anesthetic with a contrast was used for the right ONB. All patients underwent spinal or general anesthesia before the ONB. We expected that the success rate of the injection into the obturator canal would be affected by the patients’ physique, based on a report demonstrating a correlation between the depth of the obturator nerve and body mass index (BMI) [19]. The General Clinical Research Center of our institute was requested to perform a random allocation, using the retrospective data of 50 patients (median age: 74 years; BMI: 23.4 kg/m^2^) who underwent ONB, into one of the three ONB technique groups (PA, IA, and UMA) considering the following factors: age ≥ 75 years, age < 75 years, BMI ≥ 24 kg/m^2^, and BMI < 24 kg/m^2^. Patients with severe hepatic and renal dysfunction, those with severe thyroid disease, and those with an allergy to local anesthetic or contrast medium were excluded.

Spinal anesthesia was usually selected for TURBT; 12–15 mg of 0.5% high-density bupivacaine was injected at the level of L3–L5 using a 25-gauge, 7-cm Quincke needle (Spinocan; B. Braun, Melsungen AG, Germany). General anesthesia was administered to patients with coagulation abnormalities, those who had undergone spinal surgery, and those who specifically requested general anesthesia. Propofol and remifentanil were used to induce general anesthesia; a laryngeal mask (Proseal; Senko Medical Instruments Mfg. Co. Ltd., Tokyo, Japan) was inserted and oxygen, air, and either sevoflurane or desflurane were used to maintain anesthesia.

A Teflon-insulated, 22-gauge, 8-cm needle (Stimuplex D Ultra; B. Braun) was used for the ONB. A nerve stimulator (Stimuplex HNS 12; B. Braun) was used to detect the obturator nerve. Stimulation was initiated at a current of 2–3 mA for 0.1 ms and was gradually decreased to 0.5–1.0 mA.

For PA, a needle was inserted 2 cm inferior and 2 cm lateral to the outer border of the pubic tubercle [6]. When the puncture needle reached the inferior border of the superior ramus, the needle was directed 45 degrees laterally to the obturator foramen until the adductor magnus muscle reflex was elicited.

For IA, a needle was inserted at the midpoint of the femoral artery and just below the pubic tubercle in the inguinal crease [7]. The needle was tilted 30° in the cephalad direction until contraction of the gracilis or adductor longus muscle was elicited. Subsequently, the needle was inserted slightly laterally until contractions of the adductor magnus muscle were elicited.

**Fig. 1:**
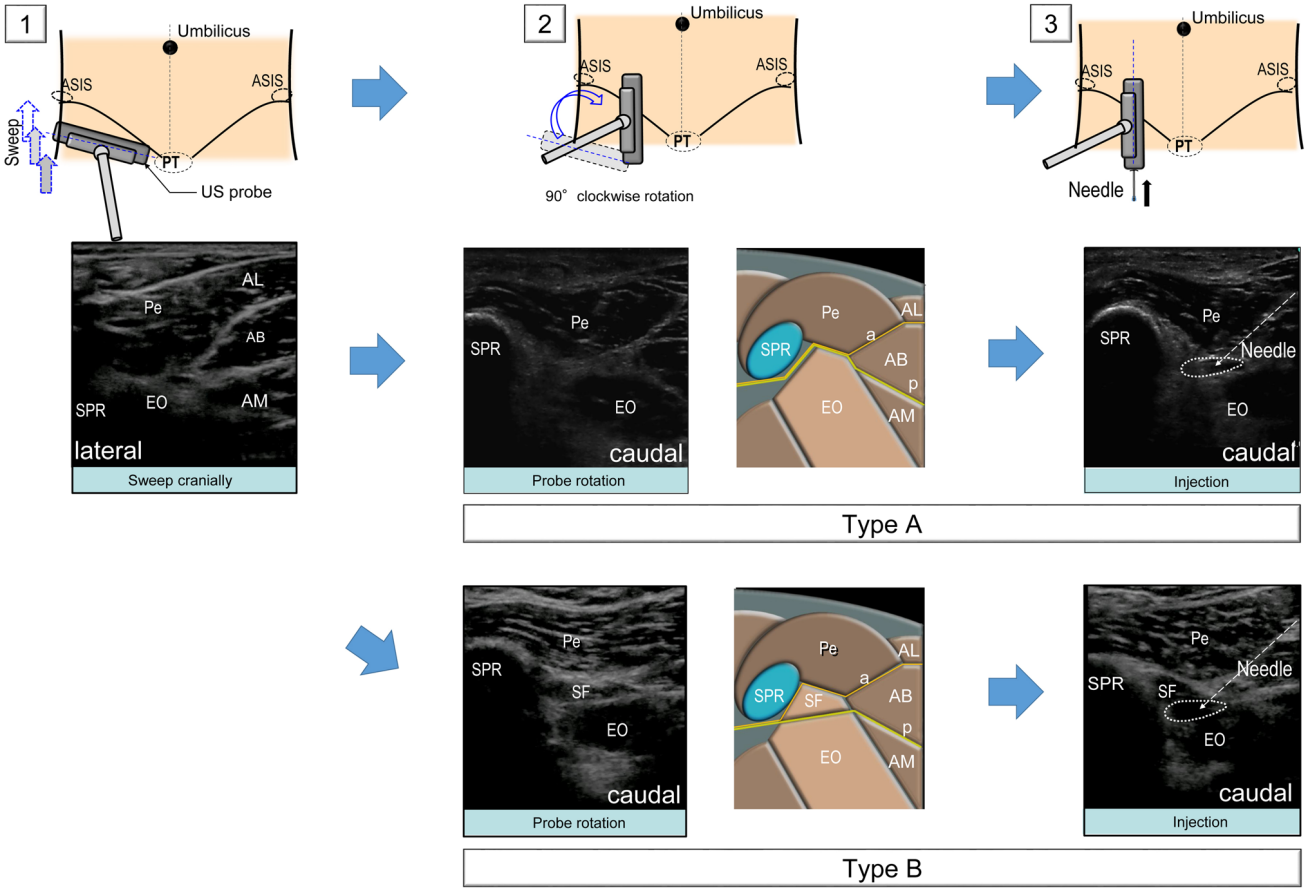
Ultrasound-guided proximal sagittal approach for the obturator nerve block. 1. Ultrasound probe is placed on the medial aspect of the inguinal crease and swept cranially. 2. External obturator muscle (EOM) is identified and the probe is rotated approximately 90°. Pattern diagrams explaining the ultrasound images are presented on the right side of each ultrasound image. The yellow line represents the course of the obturator nerve. Although the posterior branch of the obturator nerve usually passes above the external obturator muscle (type A), the branch passes between the superior and main fasciculi of the external obturator muscle in individuals with an independent superior fasciculus of the external obturator muscle (type B). The obturator nerve was identified as a centrally hypoechoic structure that is hyperechoic in the periphery of the intermuscular septum. Nerve stimulation ultimately confirmed that this structure was the obturator nerve. 3. The needle is advanced in-plane toward the posterior branch of the obturator nerve that runs in the intermuscular septum according to the pattern of the posterior branch of the obturator nerve. A mixture of local anesthetic and contrast agent was injected. *a* anterior branch of the obturator nerve, *AB* adductor brevis muscle, *AL* adductor longus muscle, *AM* adductor magnus muscle, *ASIS* anterior superior iliac spine, *EO* external obturator muscle, *p* posterior branch of obturator nerve, *Pe* pectineus muscle, *PT* pubic tubercle, *SF* superior fasciculus of the EO, external obturator muscle, *US* ultrasound

For UMA, we used the S-Nerve^®^ (FUJIFILM-SonoSite Inc., Tokyo, Japan) with a linear array probe (Fig. 1). The ultrasound probe was placed on the medial aspect of the inguinal crease and swept cranially. The external obturator muscle (EOM) was identified under the pectineus muscle, and the probe was rotated approximately 90°. Thereafter, we observed the course of the obturator nerve. Ultrasonography revealed the obturator nerve as a centrally hypoechoic structure that is hyperechoic in the periphery of the intermuscular septum. Further, a block needle was advanced towards the posterior branch of the obturator nerve and the adductor magnus muscle reflex was confirmed through stimulation.

The ONB solution (10 mL of 1.2% mepivacaine) comprised 2 mL of iohexol added to 8 mL of 1.5% mepivacaine. The block needle was fixed at the site where the adductor magnus muscle reflex was elicited; then, 5 mL of the mixed solution was injected after aspiration to detect intravascular needle placement.

A radiologist evaluated all imaging findings. After 5 mL of the mixed solution was injected, the extent of spread of the contrast medium in the obturator canal was first evaluated using X-ray fluoroscopy. In the event that 5 mL of the mixed solution was insufficient to enhance the obturator canal, additional solution was injected in 1-mL increments until the obturator canal showed up on imaging. Once the obturator canal was visible on imaging, injection of the mixed solution was discontinued, and its total volume was recorded. If the obturator canal was still not visible on imaging even after injection of 10 mL of the mixed solution, further injection of the mixture was ceased. Finally, if the obturator canal was visible with ≤ 10 mL injection of the mixed solution, it was defined as successful obturator canal enhancement.

The Institutional Review Board required us to prevent adductor reflex of the thigh during surgery—performed for patients without successful obturator canal enhancement. Therefore, for patients whose obturator canal was not visible on imaging, the tip of the needle was adjusted towards the obturator canal using X-ray fluoroscopic guidance, and additional mixed solution was injected until the obturator canal was sufficiently enhanced to prevent adductor reflex during TURBT.

The author (T.U.) performed anesthesia management, including ONB induction, for the purpose of the present study. The urologist entered the operating room after fluoroscopic evaluation of the ONB. Electrical stimulation of the bladder wall was performed near the ureteral orifice during the surgery.

### Sample size considerations

According to our preliminary examination of the 20 patients who underwent ONB using the PA or IA, 10 (50%) experienced successful injection into obturator canal with 5 mL of the mixed solution containing 2 mL of iohexol added to 8 mL of 1.5% mepivacaine. Consequently, for ONB, we expected a success rate of approximately 50% in each of the PA and IA groups, and a success rate of more than 80% in the UMA group. Therefore, the null hypothesis was that the rate of successful injection into the obturator canal using the UMA would be similar to that using the PA or IA, whereas the alternative hypothesis was that the rates of successful injection into the obturator canal would be 50% using the PA or IA and 80% using the UMA. Sample size estimation based on a significance level of 0.05 and a power of 80% revealed that we would need 39 patients in each group. With the expectation that some patients would be excluded from the study, we determined that the study should have a total sample size of 150 patients (50 in each group).

### Statistical analyses

Statistical analysis was performed using SPSS Statistics 22.0 for Windows (IBM Corporation, Chicago, IL, USA). Shapiro–Wilk test was used to test the normality of variable data in the preliminary analysis. To compare the height of the patients in multiple independent groups, a one-way analysis of variance was used. The non-parametric Kruskal–Wallis test was used to compare the age, weight, and BMI of patients in multiple independent groups. All tests were two-tailed. Fisher’s exact test was used to compare the type of anesthesia and American Society of Anesthesiologists physical status. Two-tailed *P* < 0.05 was considered significant. Fisher’s exact test was used to compare the rates of successful obturator canal enhancement among the three ONB techniques. During post hoc comparisons after Fisher’s exact tests, *P* values were corrected using Bonferroni’s method for multiple pairwise tests. Fisher’s exact tests were performed to compare the number of failed attempts to enhance the obturator canal using two ONB techniques. *P* < 0.05 (95% confidence interval) was considered statistically significant.

## Results

**Fig. 2:**
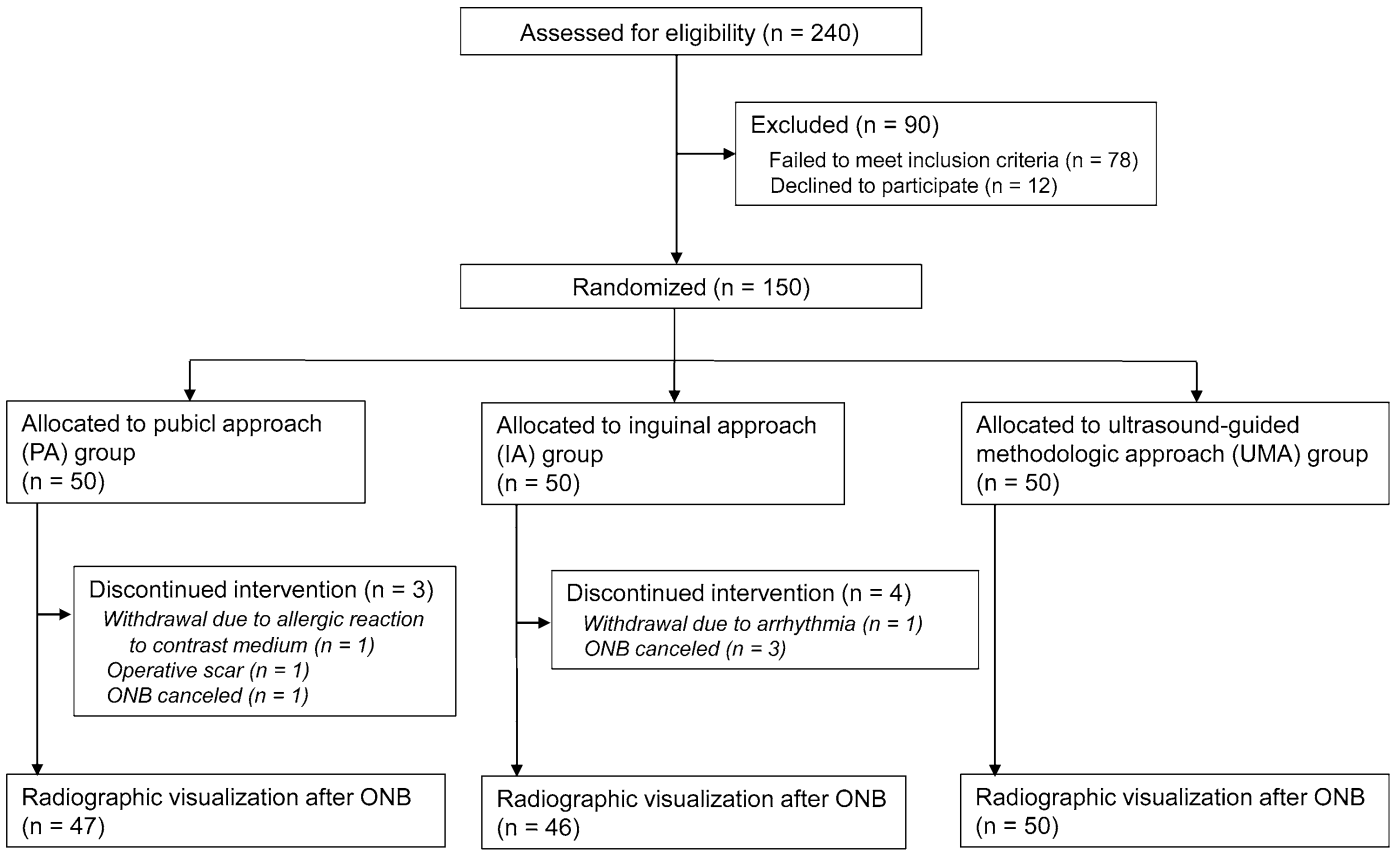
Trial diagram

A total of 150 enrolled patients were randomly allocated to three groups (PA, IA, or UMA; *n* = 50 in each group). Seven patients were excluded from the study because of the following reasons: an allergic reaction to the contrast medium identified after enrollment (*n* = 1), an operative scar at the planned ONB site (*n* = 1), a cancelation of the ONB procedure (*n* = 4), and arrhythmia (*n* = 1). Data of the remaining 143 patients were analyzed. Figure 2 shows a flow diagram of the study. Patient characteristics among the three groups were similar (Table 1).

**Table 1:** Patient characteristics

	PA group	IA group	UMA group	*P* value
Number of patients	47	46	50	–
Age (years)	73 (55–86)	74 (53–86)	73 (55–91)	0.993
Height (cm)	164.2 ± 6.0	166.3 ± 6.3	164.6 ± 5.5	0.188
Weight (kg)	64 (47–116)	64 (50–103)	65 (38–98)	0.703
BMI (kg/m^2^)	23 (18–40)	23(18–32)	23 (16–36)	0.762
Anesthesia				
Spinal	39 (83.0%)	38 (82.6%)	40 (80.0%)	0.927
General	8 (17.0%)	8 (17.4%)	10 (20.0%)	
ASA physical status				
ASA2	27 (57.4%)	32 (69.6%)	29 (58.0%)	0.409
ASA3	20 (42.6%)	14 (30.4%)	21 (42%)	

Data are presented as mean ± SD, median (range), or number (%)

*ASA* American Society of Anesthesiologists, *BMI* body mass index, *IA* inguinal approach, *PA* pubic approach, *UMA* ultrasound-guided methodologic approach

**Fig. 3:**
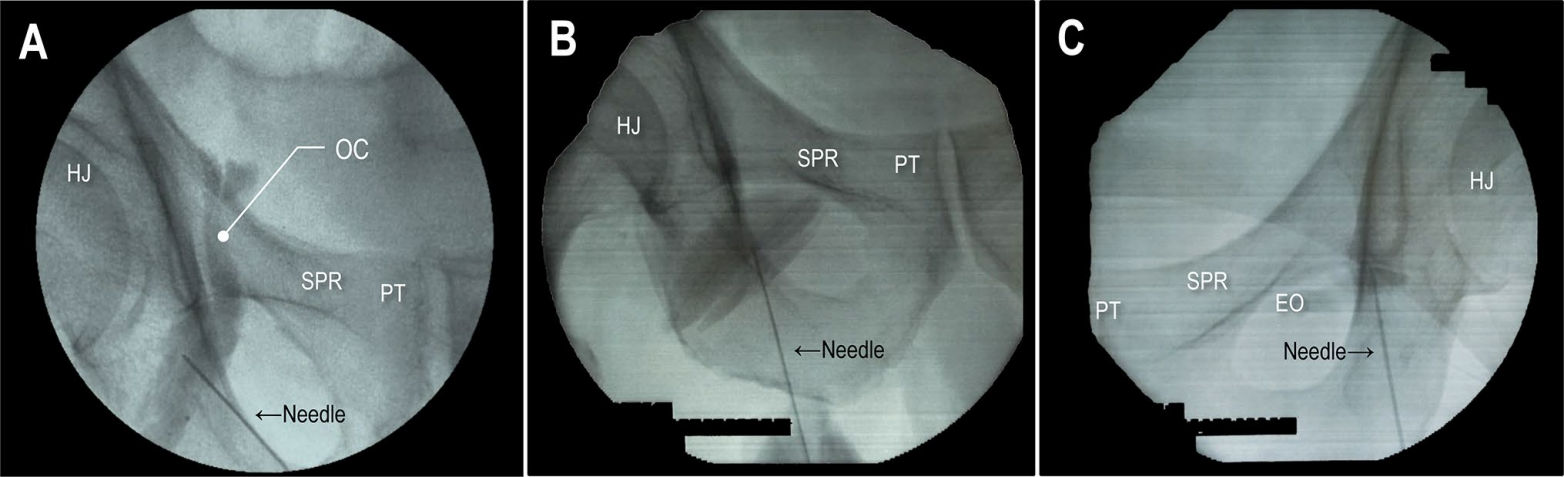
Contrast-enhanced images after obturator nerve block (ONB). Local anesthetic added to the contrast medium is injected for the ONB, and the extent of the spread of the injectate is confirmed using fluoroscopy. Contrast-enhanced image findings after the ONB could be classified into three patterns. **A** Contrast medium is detected within the obturator canal. **B** The tip of the needle is distal to the EOM, far from the obturator canal, and the injected contrast medium cannot be detected in the obturator canal. **C** As the contrast medium is injected in the superior fasciculus of the EOM, it cannot be detected in the obturator canal. *EO* external obturator muscle, *HJ* hip joint, *OC* obturator canal, *PT* pubic tubercle, *SPR* superior pubic ramus

Assessment of the primary outcome revealed that obturator canals were observed in 20 patients in the PA group (42.6%), 22 patients in the IA group (47.8%), and 42 patients in the UMA group (84%) (Fig. 3a). There was a significant difference in the success rates of obturator canal enhancement among the three groups (*P* < 0.001) (Table 2). Multiple comparisons among the three groups indicated that the success rate of obturator canal enhancement was highest in the UMA group (*P* < 0.05). Furthermore, the success rates of obturator canal enhancement did not differ between the PA and IA groups (*P* = 1.000). Enhancement of the obturator canal failed because of the following reasons: (i) block needle was positioned distal to the EOM and the mixed solution could not extend to the obturator canal beyond the EOM (*n* = 8, PA group; *n* = 15, IA group) (Fig. 3b); and (ii) superior fasciculus (SF) of the EOM was imaged but the mixed solution in the SF could not extend to the obturator canal (*n* = 19, PA group; *n* = 9, IA group; *n* = 8, UMA group) (Fig. 3c). The proportions of these reasons for failure differed significantly between the PA and IA groups (*P* = 0.026) (Table 3).

**Table 2:** Comparison of the rates of successful obturator canal enhancement between the three groups

OC enhancement	Success	Failure	Success rate	*P* value
PA group	20	27	42.6%	< 0.001
IA group	22	24	47.8%	
UMA group	42	8	84.0%	

*IA* inguinal approach, *OC* obturator canal, *PA* pubic approach, *UMA* ultrasound-guided methodologic approach

**Table 3:** Summary of the causes of obturator canal enhancement failure

	Needle tip was distal to the EO	Interference by SF	*P* value
PA group	8 (29.6%)	19 (70.4%)	0.026
IA group	15 (62.5%)	9 (37.5%)	
UMA group	0	8 (100%)	

*EO* external obturator muscle, *IA* inguinal approach, *PA* pubic approach, *SF* superior fasciculus of the external obturator muscle, *UMA* ultrasound-guided methodologic approach

After careful assessment, two types of obturator nerve ramification patterns were defined for the UMA group: A and B. In type A pattern, the anterior and posterior branches of the obturator nerve passed above the superior margin of the EOM. In type B pattern, the obturator nerve had already branched into the anterior and posterior branches when it emerged in the thigh; furthermore, the posterior branch of the obturator nerve passed between the superior and main fasciculi of the EOM (Fig. 1). A block needle was advanced to the trunk of the obturator nerve between the pectineus muscle and the EOM in cases of type A ramification pattern, for injection of the solution containing local anesthetic and contrast medium. However, in cases of type B pattern, the posterior branch of the obturator nerve between the superior and main fasciculi of the EOM was observed for injection of the solution of local anesthetic and contrast medium. Type A ramification pattern was observed in 13 of 50 patients (26%) and the obturator canal was enhanced after injection of 5 mL of the mixed solution in all 13 patients. Type B ramification pattern was observed in 37 of 50 patients (74%). In 29 of these 37 patients (78%), the mixed solution spread to the obturator canal. For patients with the type B ramification pattern, 5–7 mL of the mixed solution was necessary for enhancement of the obturator canal; however, in the remaining eight patients, the mixed solution was not injected in the intermuscular septum appropriately. Instead, the independent SF of the EOM was enhanced. Even after 10 mL of the mixed solution was injected, the obturator canal was not enhanced.

For 59 patients in whom enhancement of the obturator canal failed, the block needle was advanced using imaging guidance, and the agent containing contrast medium was directly injected in the obturator canal. Ultimately, the obturator canal was successfully enhanced in all patients, and no adductor reflexes were elicited during the procedure.

To analyze the occurrence of intravascular migration of the block needle, X-ray fluoroscopy was useful. The results revealed that the block needle tip had reached the inside of the obturator canal in six patients in the PA group and in three in the IA group, whereas the needle tip had not reached the obturator canal in the UMA group. In three of these patients in the PA group, the block needle had migrated to a blood vessel. In two of these three patients, although the aspiration test showed no backflow of blood, disappearance of the contrast medium immediately after injection revealed intravascular migration. Therefore, we suspended injection and performed the puncture again. Local anesthetic intoxication associated with intravascular injection of local anesthetic was not observed. In addition, no patients experienced nerve damage or any other organ injury.

## Discussion

We compared the success rates of a single injection of a mixed solution of a local anesthetic and contrast medium in the obturator canal among three ONB techniques. The probability of the spread of the mixed solution into the obturator canal immediately after the ONB was significantly higher with the UMA than with the PA and IA, which used a nerve stimulator.

When the ONB was administered using the nerve stimulator-guided PA and IA, the success rate of obturator canal enhancement was 50% or lower. Nerve stimulator-guided ONB techniques make it difficult to distinguish reflexes elicited by stimulation of the main branch of the obturator nerve, from some adductor reflexes elicited by stimulation of the subdivisions of the obturator nerve [12]. They also make it difficult to accurately locate the block needle tip. Consequently, in the IA group, the ONB was administered at a site distal to the obturator canal; this caused the mixed solution to flow toward the foot rather than into the obturator canal in many patients. In the PA group, although the block needle was inserted near the obturator canal in most patients, the mixed solution was injected in the SF in 19 patients.

The ultrasound-guided ONB technique was performed according to the UMA reported by Akkaya et al. [11]. The type A ramification pattern, was observed in 26% of the patients. This is almost consistent with the findings previously reported [8, 12]. For all patients with the type A ramification pattern, the obturator canal was successfully enhanced by an injection of 5 mL of the mixed solution. This suggested that for the type A ramification pattern, the mixed solution injected in the targeted obturator nerve was likely to flow in a retrograde manner into the obturator canal through the intermuscular septum, even if the volume of the mixed solution was small.

For the type B ramification pattern, the mixed solution spread into the obturator canal in 29 of the 37 patients. However, in eight patients, the mixed solution was injected in the SF, and therefore, there was no enhancement of the obturator canal. Since these eight patients had a large physique, obturator canal enhancement failure may be attributable to the difficulty in identifying the posterior branch of the obturator nerve and the failure to accurately inject the mixed solution in the intermuscular septum where the obturator nerve passed.

Previous studies on the macroscopic anatomical relationship between the course of the obturator nerve and the EOM have shown that the course of the obturator nerve in the thigh is affected by morphological changes of the SF of the EOM, which is independent from the EOM, and that the posterior branch of the obturator nerve passes between the superior and main fasciculi of the EOM in individuals with an independent SF [16, 17]. The present study demonstrated that the injection of local anesthetic in the SF could inhibit the flow of local anesthetic into the obturator canal immediately after the ONB in patients with an independent SF.

There have been reports of several other ultrasound-guided proximal approaches [11–15]. Some cadaveric studies have reported that the obturator canal and both branches of the obturator nerve were dyed through the Taha’s approach, in which 15 mL of local anesthetic was injected between the pectineus muscle and the EOM under ultrasound guidance [20], or through a unique ultrasound-guided pubic approach performed with patients in the lithotomy position [14]. The use of these ONB techniques may further increase the probability of the spread of local anesthetic into the obturator canal after the ONB in all patients, including those with an independent SF.

When ONB was administered using a nerve stimulator, the block needle reached the obturator canal in six patients in the PA group and in three patients in the IA group. However, the block needle migrated to a blood vessel in three patients in the PA group. In two of those three patients, although the aspiration test showed no backflow of blood, migration was detected because contrast imaging showed the early disappearance of the contrast medium. Insertion of a block needle in the obturator canal allows the successful blocking of both branches of the obturator nerve with a single injection; however, it is simultaneously associated with the risk of vascular puncture [18]. The present study suggested that vascular puncture might not be detected by the aspiration test alone.

The present study had several limitations. First, it was difficult to blind the anesthesiologist who performed the ONB and the patients who received spinal anesthesia. Second, for patients with failed obturator canal enhancement, the mixed solution was injected in the obturator canal under X-ray fluoroscopy to ensure the prevention of the adductor reflex of the thigh during surgery. Consequently, we were unable to assess the occurrence of the intraoperative adductor reflex of the thigh in patients with failed obturator canal enhancement after the ONB.

In conclusion, the present study suggests that a local anesthetic is more likely to spread into the obturator canal immediately after injection when the ONB is administered through the UMA than when the ONB is administered using a nerve stimulator, even in patients with an independent SF.
